# Multi-Platform Detection of Small Ruminant Lentivirus Antibodies and Provirus as Biomarkers of Production Losses

**DOI:** 10.3389/fvets.2020.00182

**Published:** 2020-04-30

**Authors:** Irache Echeverría, Ricardo De Miguel, Lorena De Pablo-Maiso, Idoia Glaria, Alfredo A. Benito, Ignacio De Blas, Damián De Andrés, Lluís Luján, Ramsés Reina

**Affiliations:** ^1^Animal Health Department, Institute of Agrobiotechnology, CSIC–Government of Navarra, Mutilva, Spain; ^2^Department of Animal Pathology, Instituto Universitario de Investigación Mixto Agroalimentario de Aragón (IA2), University of Zaragoza, Zaragoza, Spain; ^3^Molecular and Cell Biology Department, EXOPOL SL, Zaragoza, Spain

**Keywords:** small ruminant lentiviruses, diagnosis, milk production, somatic cell count, body weight, lambing size, ELISA, PCR

## Abstract

Small ruminant lentiviruses (SRLVs) are endemic in most areas of Europe, causing a chronic infection and a multisystemic disease affecting the udder, carpal joints, lungs, and central nervous system. Due to the lack of treatments and protective vaccination strategies, infection control is focused on the identification of infected animals through serological or molecular techniques. However, antigenic and genetic heterogeneity of SRLVs represent a clear drawback for diagnosis. Infected animals may present lower animal production parameters such as birth weight or milk production and quality, depending on productive systems considered and, likely, to the diagnostic method applied. In this study, four sheep flocks dedicated to dairy or meat production were evaluated using three different ELISA and two PCR strategies to classify animal population according to SRLV infection status. Productive parameters were recorded along one whole lactation or reproductive period and compared between positive and negative animals. SRLV was present in 19% of the total population, being unequally distributed in the different flocks. Less than half of the infected animals were detected by a single diagnostic method, highlighting the importance of combining different diagnostic techniques. Statistical analysis employing animal classification using all the diagnostic methods associated lambing size, lamb weight at birth, and daily weight gain with SRLV infection status in meat flocks. Milk production, somatic cell count, fat, and protein content in the milk were associated with SRLV infection in dairy flocks, to a greater extent in the flock showing higher seroprevalence. A multi-platform SRLV diagnostic strategy was useful for ensuring correct animal classification, thus validating downstream studies investigating production traits.

## Introduction

Small ruminant lentiviruses (SRLVs) cause chronic infection in sheep and goats that results in the development of a multisystemic disease that may affect animal production depending on a myriad of factors including breed susceptibility (1–3), virulence of circulating strains (4, 5), or production systems (6).

Antibody production in response to infection can be detected by serological tests, while integrated provirus in circulating monocytes can be targeted by primers in PCR strategies. Despite the important contribution of ELISA approaches in control programs established so far (7), a description of novel genotypes that enlarge antigenic heterogeneity within SRLV jeopardizes ELISA performance, leading to diagnostic failures (8–11).

Quantification of productive losses due to SRLV infection remains controversial, while some studies claim for a role of SRLV infection in decreasing quantity and quality of animal productions in both dairy and meat farms (12–14), others have revealed no differences between seronegative and seropositive animals (15–19). Slow disease development is a key feature of lentiviral infections and is the main cause of the underestimated losses in terms of animal production. However, differences in the production system, breed resistance, flock management, and parameters evaluated (20) may also explain the different associations between SRLV infection and productive traits.

Furthermore, diagnostics sensitivity may have significantly influenced studies aiming at evaluating production losses derived from SRLV infection. After initial assessment of antibody production for the detection of infected animals by agar gel immunodiffusion (AGID), later studies have demonstrated that ELISA methods are more sensitive (21). Beyond this, PCR and histopathology strategies can further improve the detection of virus in seronegative animals, indicating a benefit for combining molecular, and serological strategies (22). Antigenic and genetic heterogeneity of SRLVs are on the basis of the serologic and PCR test drawbacks, respectively. Indeed, antibodies against circulating strains are better detected using homologous antigens (8, 9, 23), and primer design is critical when developing sensitive and specific PCRs (24–26).

In this study, four sheep flocks belonging to two different production systems (dairy and meat) and breeds (Raza Navarra and Latxa Navarra) were classified as SRLV infected or uninfected using three different commercial ELISA methods, a home-made PCR kit, and a commercial PCR kit. The different commercial ELISA methods globally detected a similar number of infected animals; however, the combination of the three methods identified a significantly greater infected population in all the flocks analyzed. Furthermore, commercial PCR was more sensitive than ELISA in some cases and clearly added value to SRLV diagnosis and animal classification. Different production parameters in meat and dairy flocks were recorded during one lactation or reproductive period and, after final classification according to the different tests, were negatively (birth weight and weaning weight) or positively [somatic cell count (SCC)] associated with SRLV infection.

These results highlight the importance of using a multi-platform strategy to detect the humoral response to, as well as the presence of, different circulating strains in order to unequivocally identify infected and uninfected individuals, thereby influencing downstream studies such as production losses estimation, accreditation schemes, or control programs.

## Materials and Methods

### Animals and Samples

A total of 1,497 animals belonging to four different flocks dedicated to dairy or meat production were used (Table 1). Flocks A and B (meat flocks, focused on lamb production) and Flocks C and D (dairy flocks) were located in the north of Navarra (humid climate) and bred in a semi-extensive system with periods of housing, especially during lactation. In addition, Flock D was bred in an ecological production scheme. None of the studied animals presented clinical signs of SRLV disease.

**Table 1:** Flocks studied: location (map), breed, production system, and total sheep in each flock.

**Flock**	**Breed**	**Production system**	***n***
A	Raza Navarra	Semi-extensive meat	376
B	Raza Navarra	Semi-extensive meat	443
C	Latxa Navarra	Semi-extensive dairy	240
D	Latxa Navarra	Semi-extensive dairy	438
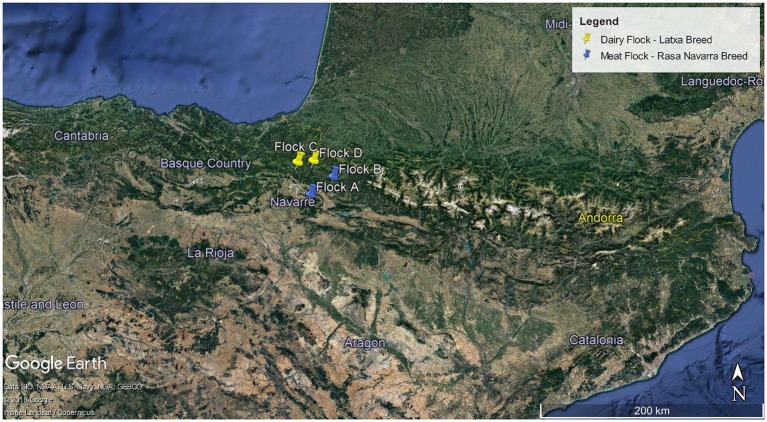			

Whole blood was obtained by jugular puncture and placed in EDTA-K^3+^ tubes. After centrifugation, plasma samples were stored at −20°C until use in ELISA. Buffy coats were washed, erythrocytes lysed, resuspended in PBS, and stored at −20°C until DNA extraction.

### ELISA Tests

Plasma samples were tested for the presence of SRLV antibodies with three commercial ELISA kits: ELISA#1 uses an Eradikit™ SRLV screening test (In3Diagnostic, Torino, Italia) that includes capsid recombinant proteins from different genotypes; ELISA#2 uses ELITEST™ MVV/CAEV (Hyphen Biomed, Neuville-sur-Oise, France) that uses recombinant a capsid protein and synthetic peptide of the TM region; and ELISA#3 uses INgezim Maedi screening™ (Ingenasa, Eurofins Technologies, Spain) that includes synthetic peptides from the Env protein. All tests were performed following manufacturers' instructions.

Data were analyzed by considering each ELISA individually and the combinations of diagnostic kits. “Total ELISA” results were built by summarizing all positive samples to at least one of the ELISAs tested. Diagnostic efficiency was determined for each of the ELISAs in samples analyzed by the three methods, in comparison with the Total ELISA result.

### DNA Extraction and PCR

Genomic DNA was extracted from buffy coat samples with and E.Z.N.A. tissue/blood kit (OMEGA, Bio-Tek, Norcross, GA, USA). DNA was quantified (NanoDrop OneC, Thermo Scientific®, Waltham, MA, USA), and real-time PCRs were performed using 250 ng of DNA (AriaMx Real Time PCR System) using the commercial kit EXOone Maedi Visna CAEV oneMix (Exopol, Spain) following manufacturer's instructions.

A total of 341 samples randomly distributed across all the flocks were analyzed by Gag PCR using primers described elsewhere (27–30). Amplicons from 28 positive reactions were sequenced for molecular characterization purposes.

An animal was finally considered as infected when at least one commercial ELISA test or one PCR method revealed a positive result (“Total Infected”).

### Productive Parameters

In meat production farms, the following factors were studied during one production period: lambing size, weaning date, birth and weaning weight, and daily weight gain (DWG).

In dairy production farms, total volume of produced milk, fat, and protein content, and SCC were parameters quantified in each monthly quality control during a whole lactation.

### Statistical Analysis

A generalized linear model (GLM) was used to assess the effect of each diagnostic strategy on each productive parameter recorded in meat flocks, taking into account the effect of the flock and the age of the mother included as covariables. Statistical results were shown as mean and standard error (SE). Production parameters were analyzed in dairy flocks by Mann Whitney's test due to significant heterogeneity in terms of SRLV prevalence, animal management, and production values between the flocks. Medians and interquartile range (IR) are shown.

The kappa coefficient was calculated to assess the agreement between tests using the methods of Cohen–Fleiss (weighted) and Pearson–Fleiss.

Statistical analyses were carried out with IBM SPSS Statistics 19.0 for Windows, and alpha error was set at 0.050.

## Results

### Serodiagnosis

Serological diagnosis strongly depended on the flock and the test used. While Flocks A, B, and C presented low seroprevalence rates of up to 10%, Flock D showed a moderate rate of seropositive animals of around 30%.

Seroprevalence values in meat flocks ranged from 2.5 to 7.5% depending on the ELISA used, showing an average value of 5% with similar ELISA efficiencies (Table 2).

**Table 2:** SRLV serological analysis of sheep belonging to meat (A and B) or dairy (C and D) flocks, employing three commercial ELISAs (1, 2, and 3).

**ELISA**	**Flock A**				**Flock B**				**Flock C**				**Flock D**			
	***n***	**Positive**		**Efficiency (%)**	***n***	**Positive**		**Efficiency (%)**	***n***	**Positive**		**Efficiency (%)**	***n***	**Positive**		**Efficiency (%)**
		***n***	**%**			***n***	**%**			***n***	**%**			***n***	**%**	
1	376	20	5.32	57.14	435	32	7.36	54.55	240	24	10	53.33	434	123	28.34	84.15
2	238	6	2.52	19.05	257	10	3.89	22.73	155	3	1.94	10.00	249	85	34.14	80.49
3	236	8	3.39	33.33	292	22	7.53	43.18	226	20	8.85	36.67	259	62	23.94	60.98
Total	376	32	8.51	–	443	52	11.74	–	240	45	18.75	–	438	142	32.42	–

*Total ELISA represents sample reactivity to any of the ELISA used. Efficiency was calculated as the ratio between reactivity to a single ELISA and the Total ELISA result*.

However, seroprevalence in dairy flocks differed not only depending on the ELISA used but also depending on the flock. While Flock C showed low seroprevalence values, up to 10% (range 1.9–10%), seropositive animals in Flock D ranged from 24 to 34.1% depending on the test used. Average values considering both flocks ranged from 14.9 to 22%. ELISA efficiencies differed between flocks and ELISA tests, ranging from 10 to 84% (Table 2).

Overall, ELISA#1 showed better results in terms of reactivity, taking into account the whole population, followed by ELISA#3 and ELISA#2. However, when considering data within flocks, ELISA#1 detected a higher number of animals in Flocks A and C, whereas ELISA#2 and ELISA#3 were more accurate in detecting infected animals in Flocks D and B, respectively. Considering samples positive to any of the kits tested (Total ELISA), a single ELISA only detected from 19 to 57% of the seropositive animals in Flock A and from 22 to 54% in Flock B; and the range in Flock C was from 10 to 53%. Single ELISA reactivity in Flock D ranged from 61 to 84.1% in comparison with Total ELISA results (Table 2).

Indeed, agreement between serological tests calculated in samples analyzed with the three methods was very good between ELISA#1 and ELISA#2 only in Flock D. Values in Flocks A and C were not calculated since no samples were found to be simultaneously positive to more than one ELISA. In addition, association was minimal or low in Flock B (data not shown).

### PCR

PCR analysis included a commercial kit that employs real-time PCR to detect and quantify different genotypes of SRLV and a home-made PCR based on previous publications. Commercial PCR detected more positive animals than did the classical home-made procedure, and both detected a total percentage of positive animals of 0, 36.8, 3.3, and 38.5% in Flocks A, B, C, and D, respectively. These values were higher than those obtained by serological methods in all flocks, except for Flock A in which the number of samples analyzed by PCR was significantly lower. In addition, an average of 10% of the seronegative animals rendered PCR-positive reactions. Consistently, PCR and ELISA agreement was minimal (Table 3).

**Table 3:** Total ELISA and PCR analyses of sheep belonging to meat (A and B) or dairy (C and D) flocks.

**Test**	**Flock A**			**Flock B**			**Flock C**			**Flock D**		
	***n***	**Positive**		***n***	**Positive**		***n***	**Positive**		***n***	**Positive**	
		***n***	**%**		***n***	**%**		***n***	**%**		***n***	**%**
Home-made PCR	55	0	0	51	8	15.68	153	5	3.27	82	10	12.19
Commercial qPCR	ND	ND	ND	86	29	33.72	ND	ND	ND	101	43	42.57
Total PCR	55	0	0	87	32	36.78	153	5	3.27	122	47	38.52
Total ELISA	376	32	8.51	443	52	11.74	240	45	18.75	438	142	32.42
Total Infected	376	32	8.51	443	59	13.32	240	50	20.83	438	148	33.11

*Total ELISA represents sample reactivity to any of the ELISAs used. Total Infected was calculated with samples positive to any of the tests used. ND, not determined*.

Sequence characterization of the Gag region in Flocks B, C, and D showed a mixed virus population between genotypes A and B2, the latter being prevalent, with high similarity in respect to previous deposited sequences. Gag immunodominant region alignment revealed no significant changes compared to sequences described (data not shown).

### Evaluation of Production in Meat Farms

Lambing size, birth body weight, weaning date, and weight gain per day were affected depending on animal classification using single or combined ELISA results as well as PCRs. Lambing size was not affected when animal classification was conducted with single ELISAs; however, a combination of the three ELISAs and Total ELISA data revealed a significant positive association. A combination of PCRs also associated SRLV infection status with higher lambing size. However, a combination of serological and molecular tests associated SRLV infection with lower lambing size (Table 4).

**Table 4:** Meat production parameters in Flocks A and B of the Raza Navarra breed according to SRLV infection status.

**Diagnosis**		**Lambing size**				**Birth weight**				**Weaning date**				**Weaning weight**				**Daily weight gain**			
		**Mean**	**SE**	***n***	***p***	**Mean**	**SE**	***n***	***p***	**Mean**	**SE**	***n***	***p***	**Mean**	**SE**	***n***	***p***	**Mean**	**SE**	***n***	***p***
ELISA#1	Positive	1.84	0.081	55	0.097	3.76	0.118	55	0.590	35.75	0.624	55	0.390	11.55	0.344	55	0.464	0.22	0.007	55	0.126
	Negative	1.97	0.023	737		3.75	0.029	730		37.89	0.236	703		11.85	0.109	678		0.21	0.002	678	
ELISA#2	Positive	1.89	0.151	19	0.586	3.77	0.212	19	0.642	36.58	1.305	19	0.793	11.40	0.712	19	0.783	0.21	0.013	19	0.860
	Negative	1.97	0.026	552		3.80	0.033	545		38.03	0.282	524		11.99	0.123	508		0.21	0.003	508	
ELISA#3	Positive	2.20	0.200	10	0.253	3.74	0.145	38	0.346	35.19	0.791	37	0.112	11.25	0.429	37	0.916	0.21	0.009	37	0.356
	Negative	1.96	0.026	561		3.77	0.033	543		38.90	0.292	514		12.15	0.127	503		0.21	0.003	503	
ELISA#1 and ELISA#2	Positive	2.2	0.2	10	0.267	3.23	0.186	10	0.007^*^	38.2	1.555	10	0.182	10.03	0.579	10	0.065	0.18	0.027	10	0.089
	Negative	1.97	0.027	571		3.81	0.033	554		37.98	0.279	533		12.00	0.122	517		0.21	0.058	517	
ELISA#1 and ELISA#3	Positive	2.11	0.261	9	0.207	3.29	0.308	9	0.013^*^	35.22	0.894	9	0.273	10.40	0.652	9	0.836	0.20	0.018	9	0.664
	Negative	1.91	0.024	574		3.78	0.032	572		37.81	0.274	542		12.12	0.124	531		0.21	0.002	531	
ELISA#2 and ELISA#3	Positive	2.33	0.333	6	0.060	3.15	0.339	6	0.007^*^	36.17	1.195	6	0.124	9.55	0.697	6	0.275	0.18	0.020	6	0.423
	Negative	1.92	0.026	464		3.83	0.036	462		38.40	0.311	438		12.05	0.136	429		0.21	0.003	429	
ELISA#1, ELISA#2, and ELISA#3	Positive	2.6	0.245	5	0.006^*^	2.90	0.281	5	0.001^*^	37.2	0.735	5	0.048^*^	8.92	0.365	5	0.124	0.16	0.011	5	0.158
	Negative	1.91	0.026	466		3.83	0.036	464		38.38	0.310	440		12.06	0.140	431		0.21	0.003	431	
Total ELISA	Positive	1.80	0.059	91	0.005^*^	3.86	0.092	91	0.399	35.68	0.519	90	0.016^*^	11.67	0.289	90	0.339	0.22	0.006	90	0.098
	Negative	1.98	0.024	700		3.74	0.029	693		38.01	0.243	667		11.85	0.112	643		0.21	0.002	642	
PCR	Positive	1.67	0.142	12	0.265	4.01	0.244	12	0.414	35.08	1.171	12	0.964	12.27	0.605	12	0.520	0.24	0.013	12	0.368
	Negative	1.80	0.069	80		3.94	0.087	78		39.96	0.759	75		12.77	0.282	71		0.22	0.007	71	
Total PCR	Positive	2.09	0.094	45	0.027^*^	3.91	0.124	45	0.916	34.93	0.625	45	0.204	10.97	0.386	43	0.992	0.20	0.008	43	0.445
	Negative	1.86	0.059	92		3.81	0.085	90		38.60	0.751	87		12.06	0.322	84		0.21	0.007	84	
Total infected	Positive	1.78	0.055	101	0.001^*^	3.90	0.087	105	0.184	35.58	0.486	100	0.034^*^	11.75	0.269	100	0.084	0.22	0.005	100	0.021^*^
	Negative	1.98	0.024	690		3.73	0.029	693		38.06	0.246	657		11.84	0.113	632		0.21	0.002	632	

*Mean, standard error (SE), and number of samples analyzed (n) of each parameter are shown. The associated probability (p) with SRLV infection status obtained in the general linearized model is indicated. Animals were classified as positive or negative by considering the following: individual ELISAs, the result obtained with the different ELISAs (i.e., ELISA#1 and ELISA#2 are samples positive to both ELISA methods), the Total ELISA and Total PCR data (samples positive to any of the ELISA or PCR tested, respectively), and the Total Infected results (obtained combining Total ELISA and Total PCR results)*.

* *p < 0.05. Bold values indicate significant differences*.

Birth weight was also associated when animals were classified according to ELISA combinations but not when considering the Total Infected result. SRLV infection influenced weaning date when considering a combination of the serological tests or the Total Infected data. Finally, gain per day was not associated when animals were classified according to single considered methods; however, the Total Infected result showed significant association with lower DWG values (Table 4).

Total ELISA-positive animals were further classified into PCR negative or positive, and production losses were evaluated (Table 5). Lower birth weight, weaning date, weight, and DWG values were observed in double-positive animals.

**Table 5:** Meat production parameters in ELISA-positive animals from Flocks A and B of the Raza Navarra breed according to PCR infection status.

**Total PCR**	**Lambing size**				**Birth weight**				**Weaning date**				**Weaning weight**				**Daily weight gain**			
	**Mean**	**SE**	***n***	***p***	**Mean**	**SE**	***n***	***p***	**Mean**	**SE**	***n***	***p***	**Mean**	**SE**	***n***	***p***	**Mean**	**SE**	***n***	***p***
Positive	2.38	0.59	16	0.000^*^	3.41	0.85	16	0.147	33.94	8.49	16	0.026^*^	9.34	2.33	16	0.001^*^	0.17	0.04	16	0.004^*^
Negative	1.75	0.33	28		3.81	0.72	28		37.58	7.37	26		12.20	2.39	26		0.22	0.04	26	

*Mean, standard error (SE), and number of samples analyzed (n) of each parameter are shown. The associated probability (p) with SRLV infection status obtained in general linearized model is indicated*.

* *p < 0.05. Bold values indicate significant differences*.

### Evaluation of Production in Dairy Farms

Classification of animals according to serological tests, individually, or collectively considered, revealed differences in SCCs between seropositive and seronegative animals (Table 6). Animal classification with both PCRs also identified elevated SCC in infected animals. Considering Total Infected animals (ELISA and PCR), SCCs were elevated in positive animals to an extent of 62% compared to uninfected individuals (*p* < 0.01). Milk yield was reduced by 6% in infected animals classified by single or combined ELISA results, as well as by commercial PCR (*p* < 0.05). Fat and protein contents were also related to SRLV serodiagnosis, while fat percentage was higher in milk from infected animals, protein content was reduced (Table 6).

**Table 6:** Milk production parameters evaluated in Flocks C and D from the Latxa breed according to SRLV infection status.

**Diagnosis**		**SCC (×10**^****3****^ **cells/ml)**				**Milk yield**				**Milk fat**				**Milk protein**			
		**Median**	**IQR**	***n***	***p***	**Median**	**IQR**	***n***	***p***	**Median**	**IQR**	***n***	***p***	**Median**	**IQR**	***n***	***p***
ELISA#1	Positive	145.25	446.4	119	0.004^*^	119	55	115	0.001^*^	6.75	1.31	119	0.001^*^	5.02	0.55	119	0.03^*^
	Negative	106.7	176.29	417		133.5	59.75	390		6.50	1.23	417		5.08	0.7	417	
ELISA#2	Positive	191.6	624.3	68	0.001^*^	117	46.5	65	<0.001^*^	6.99	0.96	68	<0.001^*^	4.9	0.46	68	0.015^*^
	Negative	104.6	160.9	261		136	65	248		6.47	1.35	261		5.07	0.62	261	
ELISA#3	Positive	133.75	616.8	67	0.165	123	59	63	0.018^*^	6.67	1.12	67	0.001^*^	5.02	0.52	67	0.181
	Negative	110.27	192.9	311		140.5	61.25	297		6.34	1.31	311		5.17	0.67	311	
ELISA#1 and ELISA#2	Positive	193.75	631.25	60	0.001^*^	117	47	59	<0.001^*^	7.05	098	60	<0.001^*^	4.91	0.45	60	0.013^*^
	Negative	104.6	161.85	269		135	63.5	254		6.47	1.33	269		5.06	0.63	269	
ELISA#1 and ELISA#3	Positive	191.6	695.6	39	0.048^*^	115	60.5	39	<0.001^*^	7.05	0.89	39	<0.001^*^	4.93	0.37	39	0.008^*^
	Negative	109.8	197.7	338		140	62	320		6.34	1.28	338		5.19	0.68	338	
ELISA#2 and ELISA#3	Positive	267	703.5	35	0.003^*^	109.5	62.75	33	0.001^*^	7.06	0.95	35	<0.001^*^	4.88	0.37	35	0.115
	Negative	106.51	161.16	246		136.5	62.5	232		6.4	5.94	246		5.07	0.66	246	
ELISA#1, ELISA#2, and ELISA#3	Positive	328.25	712.92	33	0.003^*^	115	65	32	0.002^*^	7.07	0.91	33	<0.001^*^	4.88	0.36	33	0.147
	Negative	106.33	157.94	249		136.5	63.5	234		6.40	1.38	249		5.06	0.66	249	
Total ELISA	Positive	132.35	396.05	153	0.009^*^	122.5	55.25	144	0.021^*^	6.66	1.21	153	0.010^*^	5.03	0.59	153	0.230
	Negative	106.6	170.2	383		133	60	361		6.52	1.25	383		5.08	0.71	383	
PCR	Positive	185.7	180.74	12	0.216	146	60.75	11	0.216	6.87	0.97	12	0.008^*^	5.25	1.1	12	0.954
	Negative	108.75	273.3	186		153	67	175		6.15	1.39	186		5.23	0.59	186	
Total PCR	Positive	166.25	402.85	42	0.011^*^	120	48	40	<0.001^*^	6.98	0.85	42	<0.001^*^	4.95	0.55	42	0.011^*^
	Negative	108.75	267.32	188		155	69	178		6.12	1.4	188		5.23	0.58	188	
Total infected	Positive	133.75	348	166	0.005^*^	125	53	157	0.044^*^	6.67	1.13	166	0.004^*^	5.03	0.59	166	0.350
	Negative	106.32	172.4	370		133	61	348		6.48	1.29	370		5.08	0.7	370	

*Median, interquartile range (IQR), and number of samples analyzed (n) are indicated. Animals were classified as positive or negative by considering the following: individual ELISAs, the result obtained with the different ELISAs (i.e., ELISA#1 and ELISA#2 are samples positive to both ELISA methods), the Total ELISA and Total PCR data (samples positive to any of the ELISA or PCR tested, respectively), and the Total Infected results (obtained combining Total ELISA and Total PCR results). SCC: somatic cell count. Mann–Whitney's test associated probability (p) is indicated*.

* *p < 0.05. Bold values indicate significant differences*.

Total ELISA-positive animals were again further classified into PCR-negative or PCR-positive animals (Table 7). While milk yield and protein content were reduced in ELISA- and PCR-positive animals, milk fat was inversely affected. A tendency to higher SCC was also observed in double-positive animals.

**Table 7:** Milk production parameters evaluated in ELISA-positive animals from Flocks C and D from the Latxa breed according to PCR infection status.

**Total PCR**	**SCC (×10**^****3****^ **cells/ml)**				**Milk yield**				**Milk fat**				**Milk protein**			
	**Mean**	**SE**	***n***	***p***	**Mean**	**SE**	***n***	***p***	**Mean**	**SE**	***n***	***p***	**Mean**	**SE**	***n***	***p***
Positive	660.19	156.68	28	0.084	109.54	6.00	26	0.015^*^	6.91	0.15	28	0.008^*^	4.99	0.07	28	0.111
Negative	474.97	99.45	53		138.45	6.95	49		6.39	0.16	53		5.07	0.08	53	

*Median, interquartile range (IQR), number of samples analyzed (n) and Mann–Whitney's test associated probability (p) are indicated. SCC, somatic cell count*.

* *p < 0.05. Bold values indicate significant differences*.

## Discussion

Serological diagnosis is currently the best choice for SRLV detection in livestock. It has been widely applied in control programs but also in downstream studies evaluating production losses (21, 31) or genetic susceptibility and resistance to lentiviral infection (32). However, serological methods may fail at detecting the whole infected population due to virus antigenic diversity (10, 33, 34) or to delayed seroconversion (35), encouraging the update of existing serological methods to new variants and challenging the development and evaluation of molecular methods.

In this study, we analyze an ovine population of ~1,500 individuals by ELISA methods detecting antibodies that recognize different antigen preparations. Our results demonstrate that care should be taken when ELISA tests are considered individually, since the combination of tests is able to increase the detection of seropositive animals up to 50%. In addition, we included the evaluation of a recently developed commercial qPCR that showed better detection of infected animals when compared with individual ELISA results (Table 3). These results demonstrate that the truly infected population cannot be assessed by using a single ELISA strategy or even when applying three different commercial ELISA tests, since about 10% of infected animals remained seronegative but provirus positive and detected by PCR. SRLV seroprevalence in animals varied substantially when applying just one of the herein studied ELISA methods ranging from 2 to 34% of positive animals. A combination with PCR results, either commercial or home-made, enriched the infected population in different percentages depending on the flock studied, further modifying animal classification.

Three of the analyzed flocks, two of the Rasa Navarra breed (Flocks A and B) intended for meat production, and one milk flock of the Latxa Navarra breed (Flock C) presented low seroprevalence values. However, the remaining milk flock from the Latxa Navarra breed presented a moderate seroprevalence of around 30% excluding breed or production system influence.

Antigenic heterogeneity of SRLVs seems to be at the basis for this relatively low individual sensitivity. Consistently, the antigenic preparations included in the different ELISAs used may account for this different performance, since antigenic spectrum of the circulating strains in the studied population is also a key point to consider (36). Preliminary genetic characterization of the SRLVs in the different flocks reveals the presence of a mixed virus population including strains from different genotypes and subtypes. This may explain the better performance of the ELISA test including the highest antigenic diversity.

In spite of including antigenic preparations from different genotypes, ELISA failed at detecting a proportion of infected animals that were evidenced by molecular techniques. Infection by divergent SRLV strains in these animals is unlikely since primers used in PCR were designed based on known genotypes. Instead, low or fluctuating antibody titers may account for this discrepancy between serological and molecular techniques (36, 37). Indeed, absence of serological response has been described in the periparturient period (38) as well as a result of recent infections (39). Additionally, antibody response in infected animals to viral epitopes not included in ELISAs cannot be ruled out. Therefore, the previously suggested combination between ELISA and PCR to really achieve a “gold standard” (20, 21) is reinforced from these results.

The use of more than one diagnostic technique allowed the evaluation of different animal classifications according to single ELISA, combined Total ELISA results, and the combination of these with PCRs, resulting in a Total Infected classification. Proper classification enabled determination of the real effect SRLV infection had on production traits. Of note, the use of a single ELISA may represent the detection of roughly the 61–84% of the seropositive animals as shown in Flock D of moderate seroprevalence. Furthermore, in low-seroprevalence flocks, a single ELISA may only detect 10% of the seropositive animals. ELISA performance differed among flocks, while results from Flocks A, B and C showed low seroprevalence and ELISA efficiency; in Flock D, seroprevalence and efficiency reached 32 and 84%, respectively. Breed, age, production system, nutrition, or animal management cannot be argued as important factors, since Flock C shared these features. Instead, circulation of a more prototypic SRLV in Flock D could explain this better performance.

In meat flocks, SRLV influence on animal production was clearly evidenced when serological tests were combined between them or with molecular tests. Furthermore, different interpretations could be reached, taking into account the different animal classifications. Total Infected animals, including PCR and ELISA, showed lower lambing sizes and a trend to higher birth body weight. Despite one variable possibly being related to the other, since higher lambing size implies lower birth weights (40), previous studies relate SRLV with lower birth weights (18) or rather did not find association (16, 17, 19) likely due to the low epidemiologic importance of natural *in utero* transmission (41). In contrast, serological methods associated SRLV seropositivity with lower birth body weight and with lambing size depending on the data analysis performed. While positive animals to the three ELISAs used (ELISA#1, ELISA#2, and ELISA#3) showed higher lambing size, ELISA combination (Total ELISA) associated lower lambing size with SRLV positivity. Since Total PCR results were in accordance with higher lambing size in positive animals, inclusion of ELISA false-positive reactions in the Total ELISA and Total Infected groups may help to explain this discrepancy. Previous studies including one of the ELISAs used, reported specificity values ranging from 98.4 to 99.8% with respect to AGID (42), further supporting this hypothesis.

Despite the very low seroprevalence observed in meat flocks to single ELISAs, a moderate presence of infected animals (~30%) was evidenced by PCR. Thus, PCR analysis has improved the results presented here due to detection of incipient infections that may mask SRLV influence. Chronic infections and especially SRLV show a long asymptomatic period in which ewe's body condition may inadvertently diminish, likely determining a reduced nutrient transfer to the fetus (43, 44). Sustained immune response in these infections may also alter the metabolism to a more catabolic profile, thereby reducing disposable input for the lamb. Actually, HIV infection has severe impact on pregnancy outcomes such as low birth weight and preterm delivery (45–47).

In dairy flocks, the application of single ELISA already identified higher SCC and fat content as well as lower milk yield and protein in milk from SRLV-seropositive sheep. A combination of ELISAs and PCR further confirmed this finding. Total Infected animals showed lower milk production (up to 3%) and elevated SCCs (60% increment). Augmented SCC has been already linked to SRLV infection due to epithelial cell desquamation derived from microscopic alterations in the mammary gland (48, 49) and may represent lower milk quality and, beyond, economic losses to farmers due to penalties. In the absence of clinical signs, increased SCC could be related to systemic incipient lesions that may be present in up to 20% of infected animals (50). Interestingly, recent studies show that up to 90.9% of naturally SRLV-infected animals exhibit minimal to moderate lesions in the mammary gland, this prevalence being even higher in intensive milk-producing systems (22). Increased fat content in the milk could be the simple consequence of lower production (51). Decreased protein content was found in infected animals, further pointing out SRLV influence on milk production parameters.

Among ELISA-positive animals, the PCR-negative population showed lower production losses as compared to PCR-positive animals in meat and dairy flocks. Higher viral load implies higher PCR sensitivity as well as increased disease severity (52–54). These results suggest that antibodies revealed in ELISA may play a protective role, thereby reducing clinical signs and production losses. In agreement, the presence of antibodies against SRLV in milk may reduce proviral load detection in milk cells (55).

Interestingly, lower weight at weaning presented by lambs from seropositive ewes in meat farms could be explained by the lower milk production observed in infected sheep from dairy flocks. However, milk production parameters were not evaluated in meat flocks.

Exhaustive estimation of production losses derived from infections, especially those chronic, should be evaluated after proper infection status evaluation. The multi-platform strategy applied here to classify more than 1,000 animals into SRLV infected vs. uninfected enabled the analysis of different production parameters in meat and milk-oriented semi-extensive production systems. Proper diagnosis was achieved when three different ELISA methods and two different PCRs were used. Both meat and dairy flocks showed diminished production parameters in infected animals, mainly affecting birth and weaning weights as well as milk production together with an increased number of somatic cells counts. These results highlight the crucial importance of proper SRLV infection status determination in sheep production studies and help to clarify previous colliding results obtained by other authors.

## Data Availability Statement

All datasets generated for this study are included in the article/supplementary material.

## Ethics Statement

Ethical review and approval was not required for the animal study because Samples were obtained for institutional control campaings and later used in this study. Written informed consent was obtained from the owners for the participation of their animals in this study.

## Author Contributions

IE, RR, and LD were involved in processing of the samples and DNA extraction. IE, RD, and LD performed ELISA and PCR. AB, IE, and RD contributed to real-time PCR analysis. IE, RD, and ID performed statistical analysis of data. IE, RD, and RR drafted the manuscript. ID, DD, LL, IG, and RR revised the manuscript and wrote the final version. DD, LL, and RR obtained funds and coordinated and supervised the study. All co-authors approved the final version of the manuscript.

## Conflict of Interest

AB is Head of Molecular and Cell Biology Department in Exopol. The remaining authors declare that the research was conducted in the absence of any commercial or financial relationships that could be construed as a potential conflict of interest.
